# A New Method of Skin Grafting

**Published:** 1898-05

**Authors:** 


—*The Journal.*

---

### A NEW METHOD OF SKIN GRAFTING.

Dr. George T. Doolittle, of Spokane, Wash., contributes the history of a case to the *Journal of the American Medical Association*, in which he found the so-called safety razor very useful in the securing of grafts. The case reported was an ulcer of the leg of long standing, and covered about seventy-five square inches.

"We decided," he says, "to do skin grafting and use the safety razor to cut the grafts with. The first attempt was successful, and we were able to make grafts one and a half by two and a half inches and of an even thickness. Later we made large cuts, one and a half by five inches, the large growing as readily as the small, with the advantage that the larger the grafts the less secretion from the granular surface. The advantage of the safety razor over the knife or common razor was marked by the quickness and ease with which these sections were obtained. The skin from which the grafts were taken was first shaved, so the hair would not increase the liability of the grafts to stick to the dressing, then scrubbed with green soap and water and washed over with ether or alcohol.

"A few words are necessary in regard to the razor. (The Star made the best cuts.) Set the blade forward to about the edge of the guard. Grasp the razor between the thumb and first and second finger, thumb at the front of the razor, and the first and second finger pressed firmly against the back of the blade to keep it from slipping. With the left hand hold the skin tight just back of where you begin to cut; hold the blade at an angle of about forty-five degrees with the skin surface; cut toward you and against the grain; don't be afraid to bear down until you feel the blade taking hold of the skin, which will roll up on the blade as it is cut. After a little practice a strip six or eight inches long can be cut if necessary. Remove the blade and the skin will be found gathered up and the upper surface ready to spread, which is done by placing the blade flat on the granulations, holding down the last cut edge of the skin with some light instrument and withdrawing the blade. The skin will readily spread evenly over the granular surface."—*Medical Review of Reviews.*

